# Prognostic Value of Circulating Lymphocyte Subsets in Cervical Cancer Following Postoperative Radiotherapy

**DOI:** 10.7150/ijms.107392

**Published:** 2025-02-03

**Authors:** Shanshan Wang, Mengli Zhao, Zhongrong Gao, Xiaojing Yang, Yuan Lu, Jie Fu

**Affiliations:** 1 Department of Radiation Oncology, Shanghai Sixth People's Hospital Affiliated to Shanghai Jiao Tong University School of Medicine, Shanghai, China.; 2 Department of Gynecology, The Obstetrics and Gynecology Hospital of Fudan University, 419 Fangxie Rd, Shanghai 200011, China.

**Keywords:** lymphocyte subsets, natural killer cells, prognostic factors, cervical cancer, postoperative radiotherapy

## Abstract

**Background and objective:** The prognostic value of circulating lymphocyte subsets in cervical cancer patients receiving postoperative radiotherapy remains unclear. This study aimed to explore the prognostic significance of these lymphocyte subsets in this patient population.

**Methods:** Peripheral blood samples were collected from 101 cervical cancer patients prior to receiving postoperative radiotherapy. Flow cytometry was utilized to determine the proportions and absolute counts of lymphocyte subsets, including total T cells, CD4+ T cells, CD8+ T cells, natural killer (NK) cells, and B cells. The Kaplan-Meier method and Cox regression analysis were employed to estimate the overall survival (OS) and identify the key prognostic factors. Receiver operating characteristic (ROC) curves were generated to assess the predictive accuracy.

**Results:** The survival analysis indicated that patients with a decreased proportion of NK cells (*P* = 0.02) or reduced NK cell counts (*P* = 0.01) exhibited significantly poorer overall survival (OS) compared to those with higher levels of NK cells. In univariate Cox analysis, both the proportion of NK cells (*P* = 0.025; HR, 0.33; 95% CI, 0.12-0.87) and NK cell counts (*P* = 0.015; HR = 0.28) significantly influenced OS. In multivariate analysis, the proportion of CD4+ T cells (*P* = 0.02; HR, 0.08; 95% CI, 0.01-0.72) and NK cell counts (*P* = 0.08; HR, 0.11; 95% CI, 0.01-1.37) emerged as independent prognostic factors. The areas under the ROC curves (AUCs) for NK cell counts in predicting 1-, 2-, and 3-year survival were 0.66, 0.76, and 0.68, respectively. Patients diagnosed with stage IIIC1 exhibited a significant reduction in both the absolute counts and the proportion of NK cells compared to those diagnosed with earlier stages, specifically IB3 and IIA.

**Conclusions:** Our study found that pre-treatment levels of circulating NK cell counts and proportions serve as promising prognostic biomarkers for cervical cancer patients undergoing postoperative radiotherapy

## Introduction

Cervical cancer (CC) continues to impose a significant global burden, ranking as the fourth leading cause of malignancy incidence and mortality among females^1^. Postoperative radiotherapy remains the standard treatment for patients with CC exhibiting adverse pathological factors after surgery, significantly reducing recurrence and enhancing overall survival (OS). Nonetheless, approximately 30% of patients still experience poor clinical outcomes^2^. Combining radiotherapy with immunotherapy has yielded promising results in multiple clinical trials^3^. However, further research is needed to determine the optimal timing, dosage, and combination of immunotherapy and radiotherapy to improve clinical outcomes^4^. Previous studies suggest that administering immune checkpoint inhibitors (ICIs) before chemoradiotherapy (CCRT) results in better clinical outcomes compared to their use after or during CCRT for patients with CC^5^. The function of circulating immune cells prior to radiotherapy has been demonstrated as an important prognostic factor for patients with CC^5^, ^6^. Alterations in the absolute counts and composition of these circulating immune cells reflect the state of the immune system and serve as key components of anti-tumor immune defense^7^. The composition and function of circulating lymphocyte subsets are closely associated with the prognosis of various malignancies^8^, ^9^. However, research on the prognostic value of peripheral blood lymphocyte subset counts and proportions in patients with CC before adjuvant postoperative radiotherapy remains scarce.

Among the lymphocytes, B cells and T cells are central components of the adaptive immune response, while natural killer (NK) cells represent the innate immune system^10^. These lymphocyte subsets are paramount targets in immunotherapy and serve as clinical biomarkers due to their powerful anti-tumor capabilities^11^. Nonetheless, tumor cells have evolved multiple mechanisms to evade elimination by effector immune cells^12^. Previous studies have revealed that cytotoxic T cells (CD8+ T cells) and NK cells are highly enriched in cervical cancer tissue and often transition from cytotoxic to exhausted phenotypes^13^. Altered compositions of lymphocyte subsets have been observed in the peripheral blood, tumor tissues, and lymph nodes of cancer patients, reflecting varying degrees of immune suppression associated with inherent pathological characteristics and different clinical stages^14^. Circulating immune cells offer a more accessible and readily detectable approach compared to tumor-infiltrating immune cells. Accumulating evidence has shown a correlation between baseline levels of circulating lymphocyte subsets and clinical outcomes in various malignancies, despite the exact mechanisms remaining unclear^15^-^18^. Hwang *et al.* found that early changes in circulating immune cell subsets could mirror changes in immune cell populations within tumor tissues, further reflecting the efficacy of immunotherapy in non-small-cell lung cancer (NSCLC)^19^. Wu *et al.* identified a close association between clinical characteristics and the distribution of circulating lymphocyte subgroups in early-stage CC^20^.

The immune response significantly impacts the treatment outcomes of immunotherapy combined with radiotherapy^21^. Identifying immunological prognostic biomarkers for patients receiving radiotherapy is crucial. This study focuses on investigating the prognostic value of circulating lymphocyte subsets for patients with CC before receiving postoperative radiotherapy and exploring potential associations between lymphocyte subsets and clinicopathological features of patients.

## Materials and Methods

### Study Population

This retrospective cohort study included 101 patients who underwent postoperative radiotherapy at our institution from 2019 to 2020. Each patient had previously undergone a radical hysterectomy before receiving adjuvant radiotherapy. The preoperative evaluations consisted of gynecological examinations and diagnostic imaging, including chest X-rays, abdominal computed tomography (CT) scans or ultrasounds, pelvic radiographs, and enhanced magnetic resonance imaging (MRI) or whole-body ^18^F-FDG positron emission tomography (PET) scans, aimed at ruling out the presence of distant metastases. In cases where preoperative clinical staging was uncertain, patients were assigned to a lower stage. Pathological assessment took precedence over radiological and clinical findings and served as the definitive basis for staging. Patients who were initially staged with early-stage tumors (IB to IIA) and were found to have pelvic lymph node metastasis upon postoperative evaluation were reclassified to the International Federation of Gynecology and Obstetrics (FIGO) Stage IIIC1 (2018). Patients presenting with high-risk factors, including positive or close vaginal stumps, positive lymph nodes, or parametrial invasion, as well as those with intermediate-risk factors—such as interstitial infiltration, tumor size, and lymphovascular space invasion meeting the Sedlis criteria—were recommended to receive postoperative adjuvant radiotherapy or platinum-based concurrent chemoradiotherapy in this study, in accordance with the National Comprehensive Cancer Network (NCCN) guidelines. While there was no standardized protocol for brachytherapy, the current NCCN guidelines recommended external beam radiotherapy "with or without" vaginal brachytherapy for patients identified as high-risk. In this study, adjuvant brachytherapy was administered to patients whose postoperative pathology revealed positive or close surgical margins or other high-risk features, based on the clinical expertise and judgment of the treating physician. All patients received external pelvic irradiation with a dose of 1.80-2.00 Gy per fraction, totaling an average dose of 50.40 Gy, delivered using intensity-modulated radiation therapy (IMRT).

### Inclusion and exclusion criteria

Exclusion criteria for this study included: (1) Absence of baseline data; (2) Incomplete or inaccurate medical history; (3) Presence of autoimmune, cardiovascular, or severe hepatic or renal disease; (4) Follow-up duration of less than 3 months or cases of recurrence. Follow-up was conducted every three months for the first two years post-treatment and annually thereafter, through hospital and local healthcare facility reviews, outpatient visits, inpatient assessments, and telephonic consultations. The primary clinical outcome of this study was overall survival (OS), defined as the time from the initiation of radiotherapy to the time of death or the end of follow-up.

### Patient characteristics and data retrieval

Clinical and demographic characteristics were obtained from each patient prior to treatment. Data collected included age, Karnofsky Performance Status (KPS) scores, tumor stages according to the revised 2018 International Federation of Gynecology and Obstetrics (FIGO) staging system for cervical cancer, histological subtypes, serum tumor markers, and therapeutic regimens, all extracted from the electronic medical record (EMR) system.

### Flow cytometric analysis of circulating lymphocyte subsets

A Navios™ Flow Cytometer (Beckman Coulter, Marseille, France) was utilized to analyze the proportion and absolute count of various lymphocyte subsets in peripheral blood, including NK cells (CD3-CD16+CD56+), CD3+ T cells (CD3+), CD8+ T cells (CD3+CD8+), CD4+ T cells (CD3+CD4+), and B cells (CD3-CD19+). Flow cytometric analysis employed antibodies such as CD3-FITC/CD (16+56)-PE (UCHT1/3G8/N901, Beckman Coulter, Marseille, France) and CD4-PC5/CD8-PE/CD19-PC5 (13B8.2/B9.11/J3-119, Beckman Coulter, Marseille, France). Control antibodies with matching isotypes were used to determine background staining levels. Lysing and incubating with antibodies were conducted according to the manufacturer's instructions (Beckman Coulter, Marseille, France). A flow cytometry gating strategy example was employed to identify peripheral blood lymphocyte subsets (Supplementary Figure 1).

### Statistical analysis

Statistical analyses were conducted using GraphPad Prism (v10.1.2) and R (v4.3.2) software. Continuous variables with a normal distribution were represented as mean ± standard deviation. The Kruskal-Wallis test discerned statistical disparities among three or more groups, while the Mann-Whitney U test and unpaired t-test analyzed differences between two independent groups. Relationships between categorical variables were tested using the Chi-Square statistic. Pearson's correlation determined the strength of relationships between two continuous variables. The best cut-off values for the proportion and count of lymphocyte subsets were established using the maximum Youden Index at the predictive time of 36 months, employing related R packages. The Log-Rank Test and Kaplan-Meier survival curves compared overall survival differences between two groups. Univariate and multivariate Cox regression analyses were performed to ascertain whether lymphocyte subsets hold independent prognostic value. All statistical analyses with two-tailed p-values < 0.05 were considered statistically significant.

## Results

### Clinical characteristics and treatment of patients included in the study

A total of 101 cervical cancer patients met the inclusion and exclusion criteria and were included in this study (Supplementary Figure 2). With a median follow-up time of 47.80 months, the overall survival rates at 1, 2, and 3 years were 99.0%, 89.1%, and 84.2%, respectively (Supplementary Figure 3). The primary clinical pathological features and treatment regimens for all enrolled patients are detailed in Table 1. The average age at diagnosis was 52.30 years, with a standard deviation of 10.90 years. The majority of patients were at stage IIIC1, comprising 46.5% of the study population. Among all patients, 83 (82.2%) had squamous cell carcinoma, while 18 (17.8%) exhibited other histological types. Lymph node metastasis was detected in 47 patients (46.5%). Additionally, Table 1 provides a detailed range of baseline characteristics of peripheral blood lymphocyte subsets in patients with CC.

### Decreased NK cells correlated to the poorer overall survival

**Figure 1:**
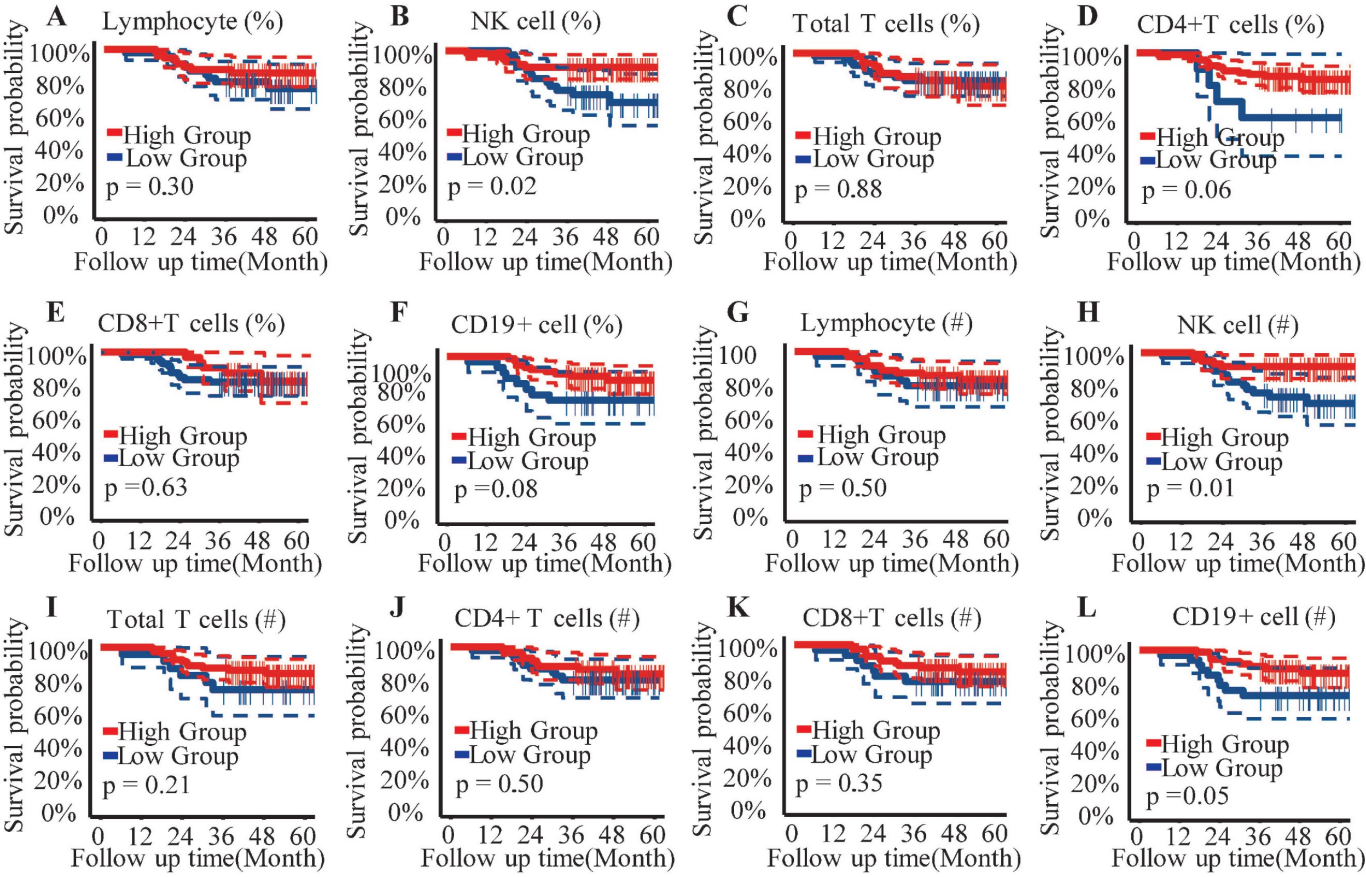
Kaplan-Meier analysis for overall survival. (A) Lymphocyte percentage; (B) Proportion of NK cells; (C) Proportion of Total T cells; (D) Proportion of CD4+ T cells subset; (E) Proportion of CD8+ T cells subset; (F) Proportion of B cells subset; (G) Absolute count of Lymphocyte; (H) Absolute count of NK cells; (I) Absolute count of Total T cells; (J) Absolute count of CD4+ T cells subset; (K) Absolute count of CD8+ T cells subset; (L) Absolute count of B cells; %, percentage of parent cells; #, absolute count.

The optimal cut-off points for circulating lymphocyte subsets were established using the Youden index (sensitivity + specificity - 1), allowing their classification into high- and low-level groups for subsequent analysis (Supplementary Table 1). Kaplan-Meier curves and the Log Rank Test assessed the prognostic value of circulating lymphocyte subsets for overall survival in patients with CC. Results from the survival analysis indicated that patients with a proportion of NK cells < 16% or an absolute NK cell count < 200/μL exhibited poorer overall survival compared to those with higher NK cell percentages or counts (Figure 1A, 1B).

### NK cells served as independent prognostic factors in cervical cancer

Univariate and multivariate Cox regression analyses identified independent prognostic factors among pretreatment circulating lymphocyte subsets. Results from the univariate Cox analysis indicated that patients with a higher percentage of NK cells (P = 0.025; HR, 0.33; 95% CI, 0.12-0.87) or a higher absolute NK cell count (P = 0.015; HR, 0.28; 95% CI, 0.10-0.78) exhibited a better prognosis in cervical cancer (Table 2). No statistically significant association with prognosis was observed for other lymphocyte subsets (total T cells, CD4+ T cells, CD8+ T cells, and B lymphocytes) in the univariate analysis (P > 0.05). Multivariate Cox regression analysis revealed that a higher proportion of CD4+ T cells (P = 0.024; HR, 0.08; 95% CI, 0.01-0.72) and a higher absolute NK cell count (P = 0.086; HR, 0.11; 95% CI, 0.01-1.37) were associated with improved prognosis (Table 2). Both NK cell count and proportion emerged as significant predictors of overall survival at 1, 2, and 3 years, with respective area under the curve (AUC) values of 0.66, 0.76, and 0.68 for NK cell count (Figure 3A) and 0.47, 0.68, and 0.69 for NK cell proportion (Figure 3B).

### Association between NK cells and clinical characteristics

**Figure 2:**
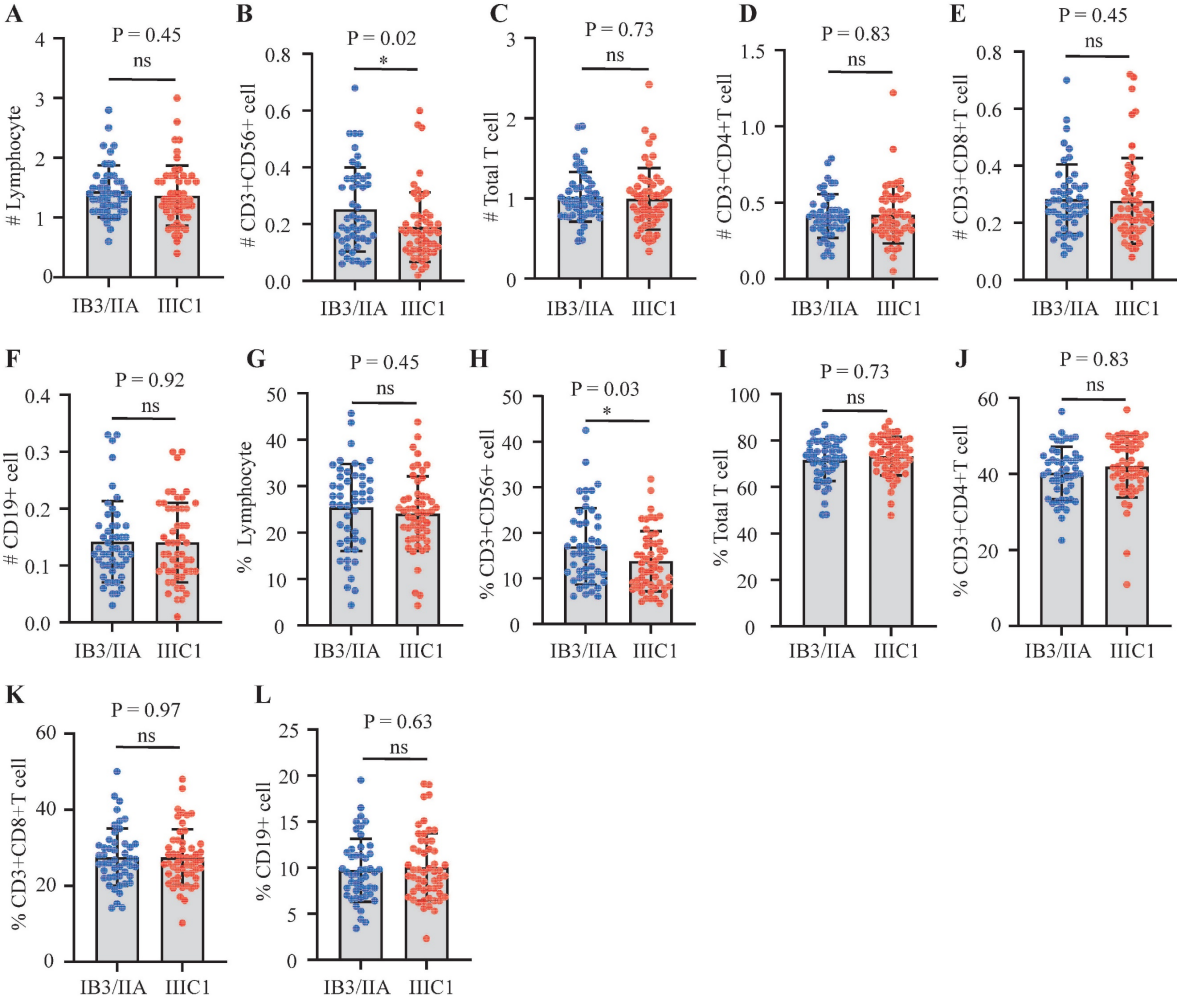
Comparing lymphocyte subsets distribution profile in CC patients with different FIGO stages (IB3/IIA1/IIA2 versus IIIC1). (A) Absolute count of lymphocyte; (B) NK cell count; (C) Total T cell count; (D) CD4+T cells subset count; (E) CD8+T cells subsets count; (F) B cells count; (G) Proportion of lymphocyte; (H) Proportion of NK cell; (I) Proportion of total T cell; (J) Proportion of CD4+ T cells subset; (K) Proportion of CD8+ T cells subset; (L) Proportion of B cell; %, percentage of parent cells; #, absolute count; NK cells, natural killer cells; Results exhibiting a P-value < 0.05 were deemed statistically significant. *P-value < 0.05; **P-value < 0.01; ***P-value < 0.001.

**Figure 3:**
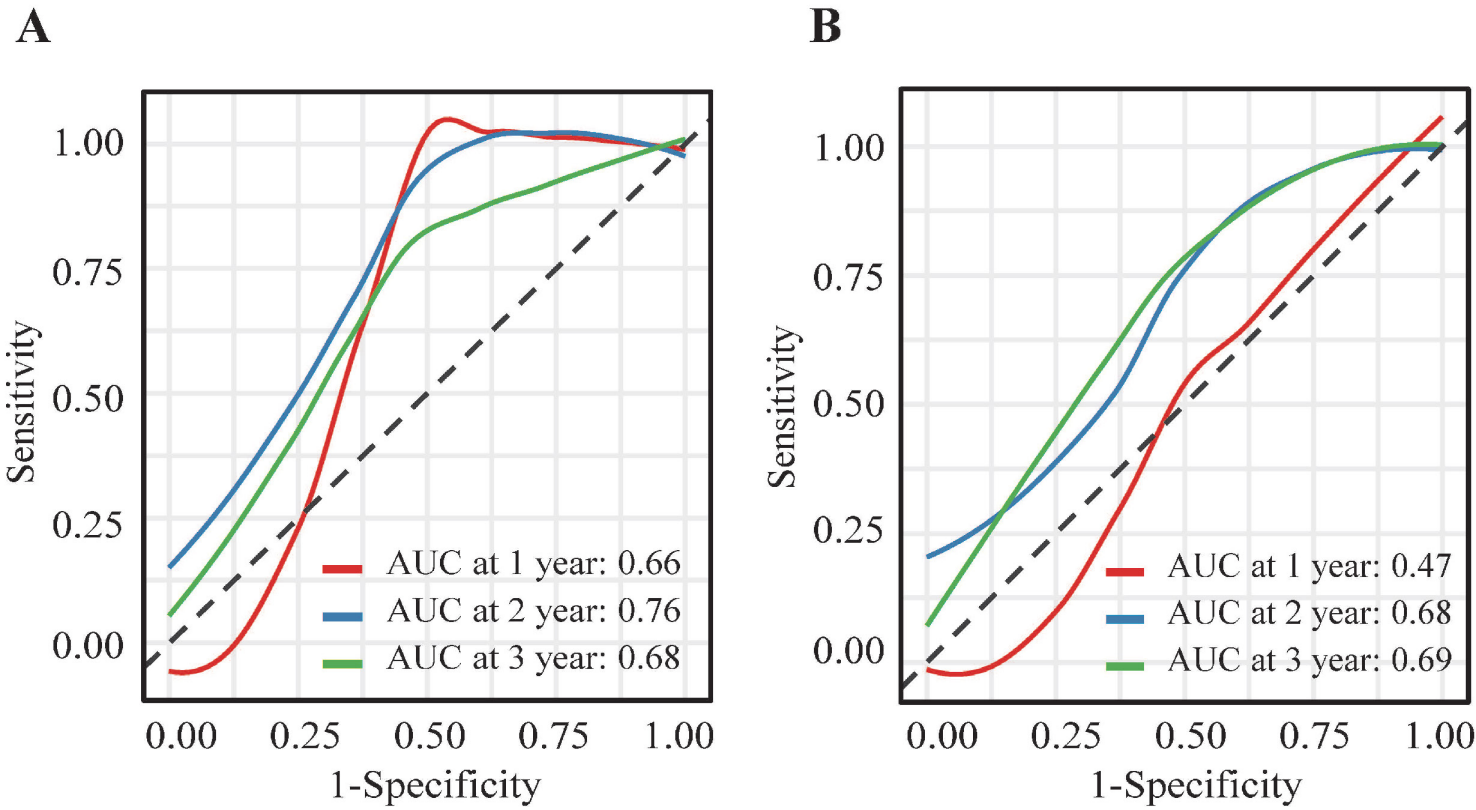
Time-ROC curves of NK cells level. (A) Time-ROC curves of absolute NK cells count at 1-, 2- and 3- year survival prediction in cervical cancer; (B) Time-ROC curves of proportion of NK cell at 1-, 2- and 3- year survival prediction in cervical cancer.

**Figure 4:**
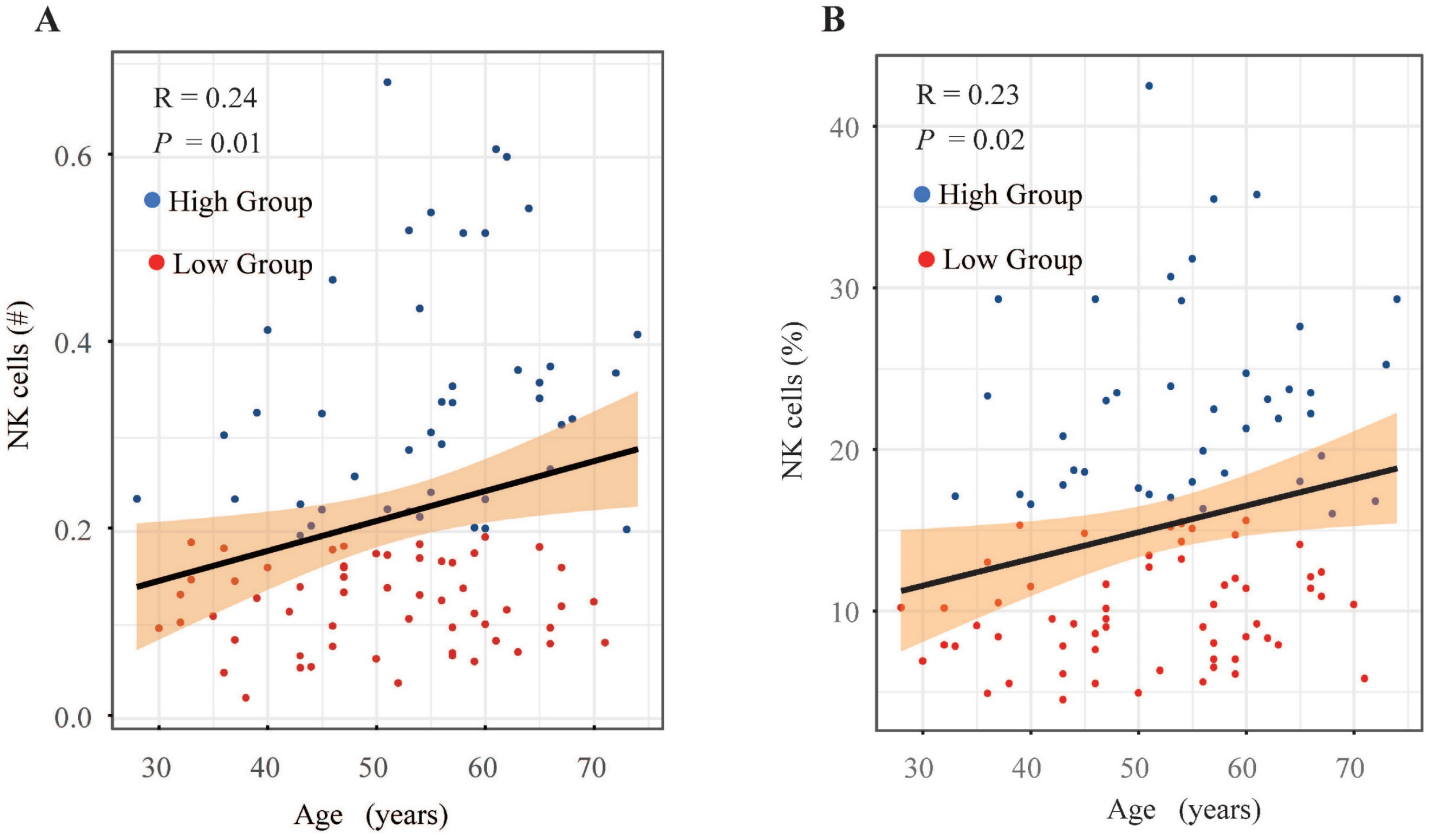
Investigation of peripheral blood NK cell levels and age-related trends in cervical cancer patients. (A) Scatter plot depicting the relationship between absolute count of NK cells and age. (B) Scatter plot depicting the relationship between the proportion of NK cells and age. #, absolute count.

A comparison was made of the distribution of peripheral lymphocyte subgroups across different FIGO stages. Patients diagnosed with FIGO stage IB3-IIA exhibited significantly higher counts (Figure 2B) and proportions (Figure 2H) of circulating NK cells compared to those with stage IIIC1. However, the proportions and absolute counts of total T cells, CD4+ T cells, CD8+ T cells, and B cells did not significantly differ among the FIGO stages (Figure 2). Associations between pretreatment levels of circulating NK cells and clinical characteristics such as age, histological subtypes, FIGO stages, pathological features, and baseline serum tumor marker levels were further analyzed. As shown in Table 3, patients with higher NK cell counts were older (average age: 54.90 years) compared to those with lower NK cell counts (average age: 50.30 years). Further analysis revealed an age-associated increase in circulating NK cells in cervical cancer (Figure 4). However, no significant differences were found between histological subtypes, serum tumor markers, and other clinical features between high and low NK cell groups (Table 3).

## Discussion

Previous studies predominantly focused on evaluating the impact of (chemo)radiotherapy on the proliferative dynamics and immunological functionality of immune cells in cervical cancer (CC). Conventional (chemo)radiotherapy-induced lymphocytopenia and immunosuppression potentially hindered its synergy with immunotherapy in CC^22^, ^23^. A prospective study by Li *et al.* indicated that administering immune checkpoint inhibitors before radiotherapy could enhance efficacy in CC^5^. Assessing the baseline immune profile before radiotherapy is critical for understanding and predicting the success of immunotherapy combined with radiotherapy in CC.

NK cells have emerged as a promising target for immunotherapy in CC^24^. This study identified a significant correlation between lower NK cell levels and poorer OS in patients with CC before postoperative radiotherapy. Patients with higher NK cell counts or proportions demonstrated significantly better prognoses (Figure 1B, 1H). Notably, advanced-stage patients exhibited decreased absolute counts and proportions of circulating NK cells, a trend reported across various cancers^25^-^28^. Consistent with previous findings, this study observed a significant reduction in both the proportion and absolute count of NK cells in patients with stage IIIC1 compared to those at stages IB3-II (Figure 2B, 2H). Wu *et al.* reported that patients older than 47 years had a higher percentage of NK cells in peripheral blood mononuclear cells (PBMC) compared to younger patients with early-stage CC^20^. Similarly, this study revealed an age-associated increase in NK cells in patients with CC (Figure 4). However, further research indicates that the age-related increase in NK cells is accompanied by a decline in their anti-tumor function, including reduced cytokine secretion and diminished cytotoxicity against target cells^29^. Tumor-induced exhaustion of NK cells, characterized by altered phenotypes and impaired antitumor functions, has been documented in various cancers^30^. Cervical cancer cells can stimulate the overexpression of suppressive cytokines while inhibiting activating ones, thereby downregulating NK cell activation and proliferation^24^, ^30^. A significant reduction in the proportion, absolute count, and cytotoxic ability of circulating NK cells has been observed in patients with CC compared to healthy controls^6^, ^31^. Garcia-Iglesias *et al.* found that dysfunctional circulating NK cells in cervical cancer exhibited downregulated expression of activating receptors of natural cytotoxicity receptors (NCRs) and NKG2D^31^. The prognostic value of infiltrated T and NK cells has been reported in various cancers^32^. Research by Wu *et al.* suggested a higher abundance of mature, cytotoxic CD56dim NK cells in peripheral venous blood, while an increased presence of immature CD56bright NK cells was noted in cervical tumor tissue^20^, ^33^. Cao *et al.* further demonstrated that intratumoral NK cells transition from cytotoxic to exhausted phenotypes, correlating with poor prognosis in CC^13^. Overexpressed TGF-β1 in cervical cancer tissues contributes to the transformation of tumor-infiltrating NK cells (TINKs) into decidual NK cells (dNKs), which are known for their pro-angiogenic function and impaired anti-tumor activity^34^, ^35^. Reversing NK cell exhaustion within the tumor microenvironment remains a challenge. Enhancing the immune surveillance and clearance functions of peripheral circulating immune cells, particularly NK cells, is essential for improving the long-term prognosis of patients with CC.

### Limitations

This study had several limitations. The sample size, particularly for subgroup analyses, was restricted. The retrospective design introduced potential selection bias. Furthermore, although radical concurrent chemoradiotherapy has been recommended as the standard treatment for FIGO 2018 Stage IIIC1 by international guidelines^36^, some patients with Stage IIIC1 opted for more precise and personalized radical surgical treatment after comprehensive evaluation, followed by adjuvant radiotherapy or chemotherapy. Additionally, due to the inherent limitations of the staging methods for Stage IIIC, discrepancies may exist in the false positive and false negative rates of pathological and imaging assessments. The lymph node status of patients undergoing radical surgery relies on preoperative imaging and gynecological examinations; consequently, a portion of patients in this study were reclassified as FIGO 2018 Stage IIIC1 based on postoperative pathological reports and subsequently received adjuvant radiotherapy or concurrent chemoradiotherapy. Future prospective or retrospective studies with larger patient cohorts and multi-center participation are necessary to conduct more detailed stratified analyses to validate the role and mechanisms of peripheral circulating immune cells in cervical cancer patients receiving adjuvant radiotherapy across different stages. Key clinical outcomes, such as distant metastasis-free survival, were not measured. Additionally, the expression of PD-1, CTLA-4, and Tim-3, indicative of exhausted immune cells, was not evaluated. Different methodologies could yield varying cut-off values for peripheral lymphocyte subsets, potentially impacting the results.

## Conclusions

This study identified pretreatment absolute counts and proportions of circulating NK cells as significant prognostic factors for patients with CC receiving postoperative radiotherapy. Strategies aimed at enhancing the function and counts of peripheral circulating NK cells hold promise for improving patient prognosis. Further research is needed to elucidate the roles of NK cells in cervical cancer.

## Supplementary Material

Supplementary figures and table.

## Figures and Tables

**Table 1:** Characteristics of Cervical Cancer Patients

Clinical Characteristics	All patients (n=101)
Age at cervical cancer diagnosis, median (IQR), y	54 (44-60)
KPS scores, no. (%)	
< 80	1 (1.0%)
≥ 80	100 (99.0%)
FIGO staging (Version 2018), no. (%)	
IB3	20 (19.8%)
IIA1	19 (18.8%)
IIA2	15 (14.9%)
IIIC1	47 (46.5%)
Histological type, no. (%)	
Squamous cell carcinoma	83 (82.2%)
Others	18 (17.8%)
LVSI, no. (%)	
Positive	77 (76.2%)
Negative	24 (23.8%)
Positive Margins, no. (%)	
Yes	9 (8.9%)
No	92 (91.1%)
Lymph node metastases, no. (%)	
Yes	47 (46.5%)
No	54 (53.5%)
EBRT technique, no. (%)	IMRT (101, 100%)
EBRT dose (Gy), median (IQR)	50.4 (45-66.4)
Percentage of lymphocyte, median (IQR)	25.2% (18.7%-31.0%)
Percentage of NK cells, median (IQR)	14.1% (9.2%-19.6%)
Percentage of Total T cells, median (IQR)	73.5% (67.6%-79.4%)
Percentage of CD4+ T cells, median (IQR)	41.3% (36.5%-46.8%)
Percentage of CD8+ T cells, median (IQR)	26.3% (22.5%-31.0%)
Percentage of B cells, median (IQR)	9.5% (7.4%-12.0%)
Lymphocyte count (cells /μL), median (IQR)	1300 (1100-1600)
NK cells count (cells /μL), median (IQR)	182 (120-306)
Total T cells count (cells /μL), median (IQR)	960 (786-1147)
CD4+T cells count (cells /μL), median (IQR)	399 (320-516)
CD8+T cells count (cells /μL), median (IQR)	251 (190-326)
B cells count (cells /μL), median (IQR)	126 (94-186)

**Abbreviations:** KPS, Karnofsky performance status; FIGO, International Federation of Gynecology and Oncology; LVSI, lymph-vascular space invasion; LNM, lymph node metastases; EBRT, external beam radiotherapy; IMRT, intensity-modulated radiation therapy.

**Table 2:** Univariable and Multivariable Cox Regression Analysis

Characteristics	Univariable Cox Regression		Multivariable Cox Regression	
	**HR (95% CI)**	***P*-value**	**HR (95% CI)**	***P*-value**
Age at cervical cancer diagnosis, y	0.99 (0.95-1.04)	0.749	0.96 (0.91-1.02)	0.195
FIGO staging (Version 2018)				
IB3	Reference		Reference	
IIA1	0.71 (0.12-4.25)	0.707	1.36 (0.11-16.18)	0.809
IIA2	1.23 (0.25-6.10)	0.799	0.62 (0.06-6.25)	0.685
IIIC1	1.48 (0.41-5.37)	0.555	0.48 (0.06-4.04)	0.502
Histological type				
Others	Reference		Reference	
Squamous cell carcinoma	0.76 (0.25-2.30)	0.621	0.29 (0.06-1.46)	0.134
LVSI				
Negative	Reference		Reference	
Positive	2.83 (0.65-12.34)	0.165	1.28 (0.22-7.52)	0.782
Positive Margins				
No	Reference		Reference	
Yes	2.83 (0.65-12.34)	0.165	1.28 (0.22-7.52)	0.782
Lymph node metastases	1.18 (0.98-1.41)	0.133	1.17 (0.95-1.45)	0.210
No	Reference		Reference	
Yes	2.60 (0.93-7.30)	0.070	4.24 (0.44-41.29)	0.213
Percentage of lymphocyte				
Low Group	Reference		Reference	
High Group	0.63 (0.25-1.59)	0.326	0.53 (0.12-2.34)	0.400
Percentage of NK cells				
Low Group	Reference		Reference	
High Group	0.33 (0.12-0.87)	0.025	0.30 (0.03-3.16)	0.315
Percentage of Total T cells				
Low Group	Reference		Reference	
High Group	1.07 (0.42-2.70)	0.885	0.35 (0.06-2.08)	0.249
Percentage of CD4+ T cells				
Low Group	Reference		Reference	
High Group	0.33 (0.11-1.02)	0.054	0.08 (0.01-0.72)	0.024
Percentage of CD8+ T cells				
Low Group	Reference		Reference	
High Group	0.78 (0.28-2.19)	0.635	0.32 (0.06-1.85)	0.205
Percentage of B cells				
Low Group	Reference		Reference	
High Group	0.44 (0.17-1.11)	0.083	0.22 (0.03-1.51)	0.125
Lymphocyte count (cells /μL)				
Low Group	Reference		Reference	
High Group	0.72 (0.28-1.87)	0.504	3.30 (0.34-32.17)	0.304
NK cells count (cells /μL)				
Low Group	Reference		Reference	
High Group	0.28 (0.10-0.78)	0.015	0.11 (0.01-1.37)	0.086
Total T cells count (cells /μL)				
Low Group	Reference		Reference	
High Group	0.54 (0.20-1.44)	0.217	0.33 (0.03-3.13)	0.332
CD4+T cells count (cells /μL)				
Low Group	Reference		Reference	
High Group	0.73 (0.29-1.84)	0.503	4.15 (0.62-27.85)	0.142
CD8+T cells count (cells /μL)				
Low Group	Reference		Reference	
High Group	0.64 (0.25-1.64)	0.350	0.52 (0.06-4.61)	0.559
B cells count (cells /μL)				
Low Group	Reference		Reference	
High Group	0.41 (0.16-1.04)	0.059	0.85 (0.10-7.63)	0.886

**Abbreviations:** KPS, Karnofsky performance status; FIGO, International Federation of Gynecology and Oncology; LVSI, lymph-vascular space invasion;

**Table 3:** Associations Between Peripheral Blood Natural Killer Cells and Clinical Characteristics.

	NK cells level (%)			NK cells level (#)		
**Parameters**	**High Group**	**Low Group**	***P*-value**	**High Group**	**Low Group**	***P*-value**
Age (mean ± SD)	54.8 (10.5)	50.6 (11.0)	0.061	54.9 (10.5)	50.3 (10.9)	0.034
KPS scores			0.406			0.436
< 80	1 (2.44%)	0 (0.00%)		1 (2.27%)	0 (0.00%)	
≥ 80	40 (97.6%)	60 (100%)		43 (97.7%)	57 (100%)	
Histology			1.000			0.858
Other	7 (17.1%)	11 (18.3%)		7 (15.9%)	11 (19.3%)	
Squamous	34 (82.9%)	49 (81.7%)		37 (84.1%)	46 (80.7%)	
LVSI			0.554			0.357
Negative	8 (19.5%)	16 (26.7%)		8 (18.2%)	16 (28.1%)	
Positive	33 (80.5%)	44 (73.3%)		36 (81.8%)	41 (71.9%)	
Positive Margins			0.735			1.000
No	38 (92.7%)	54 (90.0%)		40 (90.9%)	52 (91.2%)	
Yes	3 (7.32%)	6 (10.0%)		4 (9.09%)	5 (8.77%)	
LNM			0.564			0.415
No	21 (51.2%)	26 (43.3%)		23 (52.3%)	24 (42.1%)	
Yes	20 (48.8%)	34 (56.7%)		21 (47.7%)	33 (57.9%)	
CEA (mean ± SD)	1.68 (1.01)	2.60 (9.35)	0.451	1.73 (0.99)	2.61 (9.60)	0.496
CA50 (mean ± SD)	5.81 (4.17)	6.63 (10.8)	0.594	5.77 (4.24)	6.70 (11.1)	0.565
CA125 (mean ± SD)	37.8 (35.7)	32.7 (23.7)	0.424	38.3 (35.8)	32.1 (22.7)	0.320
CA153 (mean ± SD)	12.2 (8.04)	12.8 (8.09)	0.734	11.8 (7.91)	13.1 (8.15)	0.410
CA199 (mean ± SD)	11.3 (7.56)	12.1 (18.2)	0.777	11.3 (7.54)	12.1 (18.6)	0.755
SCC-Ag (mean ± SD)	1.00 (0.94)	1.11 (1.22)	0.626	1.07 (0.98)	1.06 (1.21)	0.952

**Abbreviations:** %, percentage of parent cells; #, absolute count; NK cells, natural killer cells; KPS, Karnofsky performance status; FIGO, International Federation of Gynecology and Oncology; LVSI, lymph-vascular space invasion; LNM, lymph node metastases; CEA, carcinoembryonic antigen; CA50, cancer antigen 50; CA125, cancer antigen 125; CA153, cancer antigen 153; CA199, cancer antigen 199; SCC-Ag, squamous cell carcinoma antigen. *P*-values were calculated using the Mann-Whitney test for continuous variables and χ2 test. *P*-values were calculated using the Mann-Whitney test for categorical variables.
