# Supplementary material for: Prognostic Value of Circulating Lymphocyte Subsets in Cervical Cancer Following Postoperative Radiotherapy
Source: Int J Med Sci. 2025 Feb 3;22(5):1029–38. doi: 10.7150/ijms.107392 (PMC11866531; doi:10.7150/ijms.107392)
Supplement: Supplementary file 1 — Supplementary figures and table. [file ijmsv22p1029s1.pdf]

Supplementary Material

Prognostic Value of Circulating Lymphocyte Subsets in Cervical Cancer Following Postoperative Radiotherapy

**Figure S1**	Flow cytometry gating strategy for peripheral blood lymphocyte subsets.	Page 2
**Figure S2**	Diagram of the study flow.	Page 3
**Figure S3**	Overall survival for the entire cohort.	Page 4
**Table S1**	Identification of the optimal cut-off value by using the maximum Youden index.	Page 5

Supplementary Figure 1

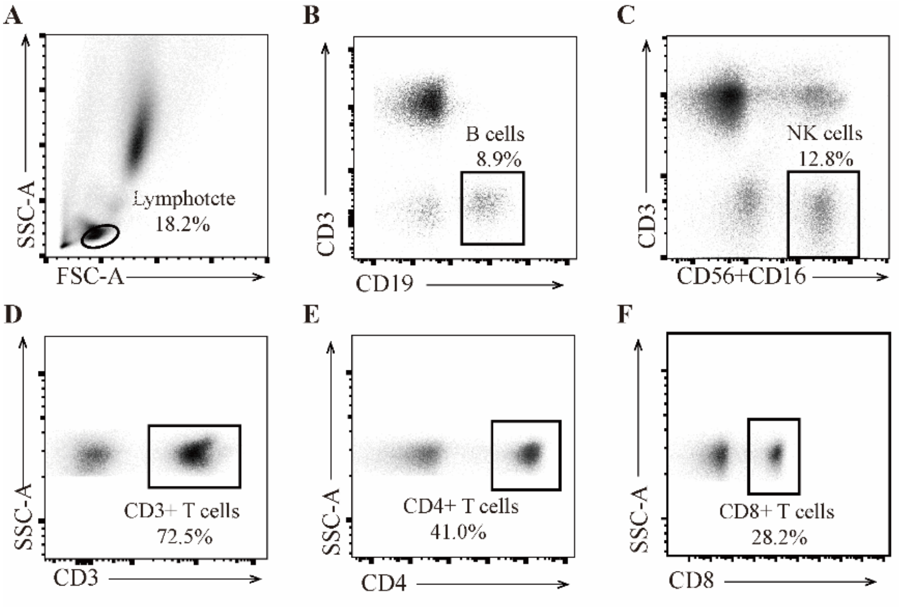

Supplementary Figure 1. Flow cytometry gating strategy for peripheral blood lymphocyte subsets. (A) Lymphocytes were selected based on forward and side scatter; (B) peripheral B cells as (CD3-CD19+) were selected; (C) peripheral natural killer (NK) cells (CD3-CD16+CD56+) were selected; (D) Total T cells (CD3+) were selected; (E) CD4+T cells (CD3+CD4+) were selected; (F) CD8+T cells (CD3+CD8+) were selected.

Supplementary Figure 2

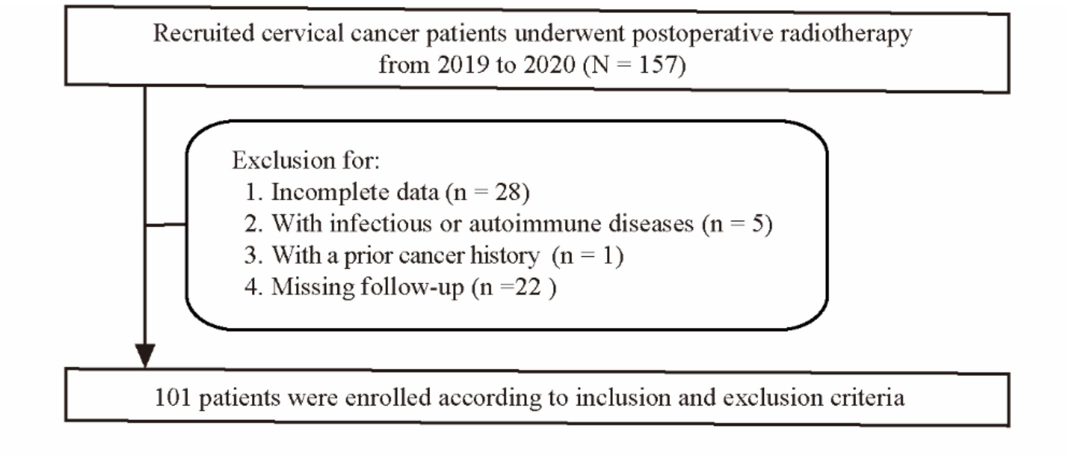

Supplementary Figure 2. Diagram of the study flow.

Supplementary Figure 3

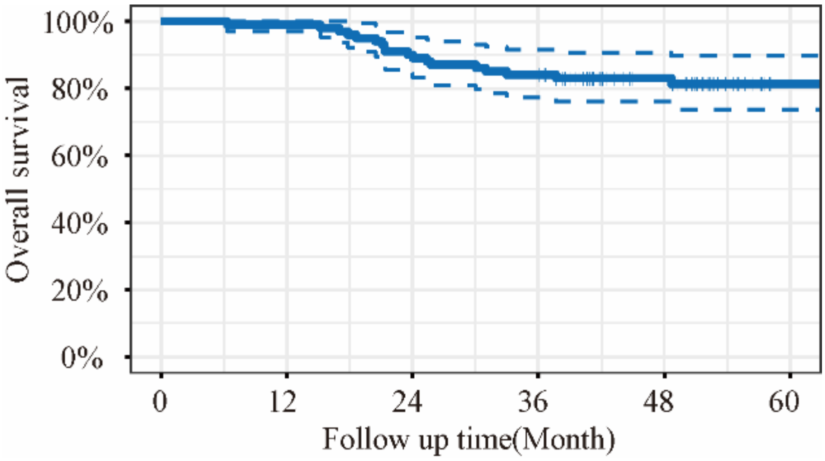

Supplementary Figure 3. Overall survival for the entire cohort.

Supplementary Table 1. Identification of the Optimal Cut-off Value Using the Maximum Youden Index.

Peripheral blood lymphocyte subsets	Cutoff value at Max Youden Index	N (%)	Prediction time (months)	AUC value at 36 months
**Percentage of lymphocyte**			36	0.54
High Group	≥ 26.20	45 (44.6%)		
Low Group	< 26.20	56 (55.4%)		
**Percentage of NK cells**			36	0.69
High Group	≥ 16.00	41 (40.6%)		
Low Group	< 16.00	60 (59.4%)		
**Percentage of Total T cells**			36	0.46
High Group	≥ 72.80	53 (52.5%)		
Low Group	< 72.80	48 (47.5%)		
**Percentage of CD4+ T cells**			36	0.51
High Group	≥ 50.00	10 (9.90%)		
Low Group	< 50.00	91 (90.1%)		
**Percentage of CD8+ T cells**			36	0.50
High Group	≥ 24.00	70 (69.3%)		
Low Group	< 24.00	31 (30.7%)		
**Percentage of B cells**			36	0.61
High Group	≥ 11.65	29 (28.7%)		
Low Group	< 11.65	72 (71.3%)		
**Lymphocyte count (cells /µL)**			36	0.58
High Group	≥ 1600	33 (32.7%)		
Low Group	< 1600	68 (67.3%)		
**NK cells count (cells /µL)**			36	0.68
High Group	≥ 200	44 (43.6%)		
Low Group	< 200	57 (56.4%)		
**Total T cells count (cells /µL)**			36	0.57
High Group	≥ 1182	23 (22.8%)		
Low Group	< 1182	78 (77.2%)		
**CD4+T cells count (cells /µL)**			36	0.56
High Group	≥ 418	44 (43.6%)		
Low Group	< 418	57 (56.4%)		
**CD8+T cells count (cells /µL)**			36	0.55
High Group	≥ 309	31 (30.7%)		
Low Group	< 309	70 (69.3%)		
**B cells count (cells /µL)**			36	0.60
High Group	≥ 165	32 (31.7%)		
Low Group	< 165	69 (68.3%)		
**Ratio of CD4/CD8**			36	0.48
High Group	≥ 1.96	30 (29.7%)		
Low Group	< 1.96	71 (70.3%)		
